# The implementation of infection prevention and control measures and health care utilisation in ACF-supported health facilities during the COVID-19 pandemic in Kinshasa, Democratic Republic of the Congo, 2020

**DOI:** 10.1080/16549716.2023.2258711

**Published:** 2023-10-17

**Authors:** Chiara Altare, Linda Matadi Basadia, Natalya Kostandova, Justus Nsio Mbeta, Sophie Bruneau, Caroline Antoine, Marie Petry

**Affiliations:** a Department of International Health, Johns Hopkins Bloomberg School of Public Health, Baltimore, MD, USA; b Johns Hopkins Center for Humanitarian Health, Baltimore, MD, USA; c Department of Health and Nutrition, Action contre la Faim, Kinshasa, The Democratic Republic of Congo; d Department of Epidemiology, Johns Hopkins Bloomberg School of Public Health, Baltimore, MD, USA; e Technical Secretariat, Multisectorial Response Committee, Ministry of Health, Kinshasa, The Democratic Republic of Congo; f Department of Operations, Action contre la Faim, Paris, France; g Technical and Advocacy Department, Action contre la Faim, Paris, France

**Keywords:** COVID-19, Infection prevention and control, Kinshasa, Democratic Republic of the Congo, Routine health services

## Abstract

**Background:**

Infection prevention and control (IPC) was a central component of the Democratic Republic of the Congo’s COVID-19 response in 2020, aiming to prevent infections and ensure safe health service provision.

**Objectives:**

We aimed to assess the evolution of IPC capacity in 65 health facilities supported by Action Contre la Faim in three health zones in Kinshasa (Binza Meteo (BM), Binza Ozone (BO), and Gombe), investigate how triage and alert validation were implemented, and estimate how health service utilisation changed in these facilities (April–December 2020).

**Methods:**

We used three datasets: IPC Scorecard data assessing health facilities’ IPC capacity at baseline, monthly and weekly triage data, and monthly routine data on eight health services. We examined factors associated with triage and isolation capacity with a mixed-effects negative binomial model and estimated changes in health service utilisation with a mixed-model with random intercept and long-term trend for each health facility. We reported incidence rate ratios (IRRs) for level change when the pandemic began, for trend change, and for lockdown and post-lockdown periods (Gombe). We estimated cumulative and monthly percent differences with expected consultations.

**Results:**

IPC capacity reached an average score of 90% by the end of the programme. A one-point increase in the IPC score was associated with +6% and +5% increases in triage capacity in BO and Gombe, respectively, and with +21% and +10% increases in isolation capacity in the same zones. When the pandemic began, decreases were seen in outpatient consultations (IRR: 0.67, 95% confidence interval (CI) [0.48–0.95] BM&BO-combined; IRR: 0.29, 95%CI [0.16–0.53] Gombe), consultations for respiratory tract infections (IRR: 0.48, 95%CI [0.28–0.87] BM&BO-combined), malaria (IRR: 0.60, 95%CI [0.43–0.84] BM&BO-combined, IRR: 0.33, 95%CI [0.18–0.58] Gombe), and vaccinations (IRR: 0.27, 95%CI [0.10–0.71] Gombe). Maternal health services decreased in Gombe (ANC1: IRR: 0.42, 95%CI [0.21–0.85]).

**Conclusions:**

The effectiveness of the triage and alert validation process was affected by the complexity of implementing a broad clinical definition in limited-resource settings with a pre-pandemic epidemiological profile characterised by infectious diseases with symptoms like COVID-19. Readily available testing capacity remains key for future pandemic response to improve the disease understanding and maintain health services.

## Introduction

The fast spreading of SARS-CoV-2 infections since the first notification in China in December 2019 triggered extensive response activities worldwide, intending to contain infections and deaths and ensure the continuation of essential health services. Already fragile health systems in many African countries were expected to have limited resources to face this additional challenge, and predictions of both direct and indirect effects of COVID-19 were dire [1,2].

The Government of the Democratic Republic of the Congo (DRC), like many other countries, quickly developed a multisectoral strategic national plan to respond to the COVID-19 epidemic [3] with the support of technical and financial partners. The national response plan aimed to ensure an effective response in the city of Kinshasa and operational readiness in the provinces not yet affected. It encompassed coordination, case management, laboratory and diagnostic capacity, infection prevention and control (IPC), risk communication and community engagement, logistics, psychosocial support, and non-pharmaceutical measures, such as physical distancing and mask-wearing [3]. Furthermore, a state of emergency was declared on 24 March 2020, for the entire country, leading to border closure, obligatory mask-wearing, hand washing, school closure, and a ban on all gatherings.

The first imported case was confirmed on 10 March 2020, in the capital, Kinshasa [4], the epidemic’s epicentre in DRC since then. The most affected areas were the health zones of Gombe, Binza Meteo, Binza Ozone, Lemba, and Limité. A lockdown was imposed in the health zone of Gombe from 6 April 2020 to 29 June 2020. Furthermore, several humanitarian actors supported the government’s response activities to enhance health providers’ capacity to prepare for and respond to the pandemic. Among them, Action contre la Faim (ACF) obtained emergency funding to strengthen IPC capacity in health facilities in the three most affected health zones (Gombe, Binza Meteo, and Binza Ozone) from March to December 2020.

In parallel to ensuring response capacity, previous outbreaks have shown that attention should be given to possible indirect effects of epidemics, such as diverting resources from routine services, reducing health service utilisation, and increasing mortality risk for causes unrelated to the outbreak [5]. Analyses from low- and middle-income countries reported extensive service disruptions, with varying duration and magnitude across services, countries, and time [6,7]. Outpatient consultations, child vaccination, and consultations for infectious diseases were the most affected services in many countries. An analysis from DRC [8] reported little to no negative COVID-19 effects on overall consultations and maternal and child health services at the national level. However, when looking at Kinshasa, they did see a drop in outpatient consultations, antenatal care, vaccinations, and consultations for infectious diseases at the beginning of the pandemic. Another analysis encompassing only Kinshasa found that outpatient consultations and services for infectious and chronic diseases suffered substantial disruptions in 2020, especially in Gombe, while maternal and child health services were not largely affected [9]. While various factors influence the individual's decision to seek care in case of illness (e.g. perceived severity and risk, proximity, trust, education, residence, financial resources, and opportunity cost [10,11]), the COVID-19 pandemic may have shifted priorities and added layers of complexities (e.g. mobility restrictions). We aim to assess the evolution of the IPC capacity of ACF-supported facilities in Kinshasa and investigate how triage and alert validation were implemented at the beginning of the COVID-19 pandemic. Furthermore, we estimate how health service utilisation has changed during the first months of the COVID-19 pandemic in the same facilities.

## Methods

### Study setting

The study focused on Kinshasa, DRC’s capital city, which hosts 12 million people. The city is divided into 35 health zones and is served by 851 health centres and 121 hospitals [9], including private and public facilities. During the study period (March–December 2020), 13,885 COVID-19 cases were reported in Kinshasa, corresponding to 78% of all cases registered in the entire country over the same period [12]. During the first 3 months of the pandemic, half of the national testing capacity was concentrated in Kinshasa (i.e. five of the ten available Gene Xpert platforms were in the capital, and the remaining five were in other provinces).

### ACF’s programme

The study covers three of Kinshasa’s most affected health zones: Gombe, Binza Meteo, and Binza Ozone, where ACF’s programme was implemented. ACF’s intervention aimed to reduce COVID-19 transmission among affected communities and in supported health facilities and to maintain essential health services. The intervention logic centred around IPC activities; strengthening the health providers’ capacity to prevent nosocomial infections would contribute to both reducing transmission and ensuring the safe provision of essential services. Implemented activities included training of health care workers on IPC and water sanitation and hygiene (WASH) measures; provision of personal protective equipment (PPE); monitoring of stocks and supply; set-up of triage pathways and temporary isolation areas in health facilities; improving waste management, water supply, and hygiene practices; and participating in coordination mechanisms.

**Figure 1.**
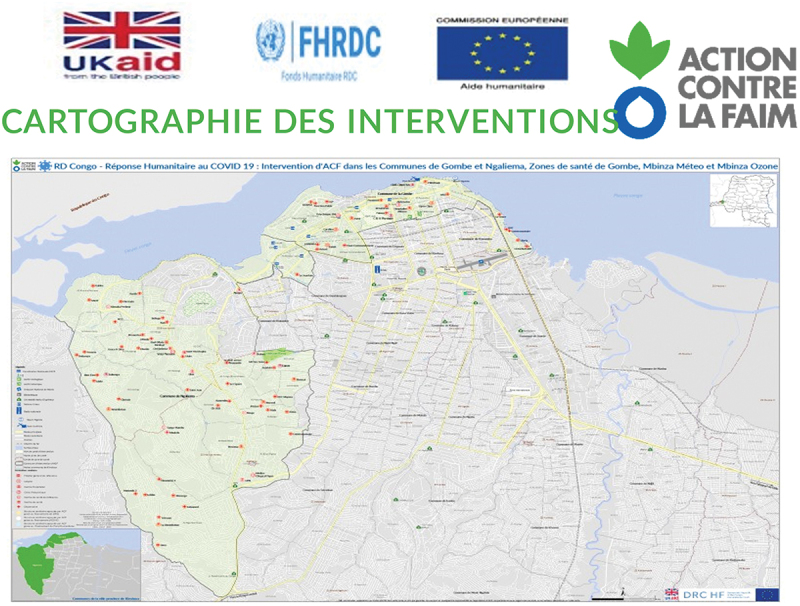
Map of Kinshasa with highlighted health zones where ACF’s intervention was implemented.

Among the 83 health facilities existing in the three health zones, 65 level III and IV health facilities were supported by ACF following a baseline IPC capacity assessment (21 health facilities in Gombe, 22 in Binza Meteo, and 22 in Binza Ozone (Figure 1)).

Supported health facilities were selected in consultations with health authorities according to the following inclusion criteria: number of monthly consultations, number of confirmed COVID-19 cases, IPC scorecard <50%, number of beds, having an established building (i.e. those with temporary structures or on short-term rentals were excluded as some interventions aimed at improving the infrastructure), and showing willingness and interest in the project. Three health facilities were excluded after being selected (one remained closed during the implementation phase, one belonged to another health zone where ACF did not intervene, and the third had to close due to financial difficulties). Therefore, three additional health facilities were included; however, they lack baseline data for IPC assessment. The project was implemented between 1 April 2020 and 15 December 2020.

### Data sources and study outcomes

#### IPC scorecard

The IPC scorecard was developed by the IPC Commission led by the DRC’s Ministry of Health (MoH) with the support of humanitarian actors, building upon the IPC scorecard used for Ebola (template available in Supplementary material section 1). The first version included 11 sections (triage and isolation, IPC organisation, hand hygiene, PPE, decontamination and sterilisation of medical equipment, decontamination of hospital linens, hospital environment, waste management, WASH infrastructure, active case search in the health facility, and communication) for a total of 44 elements to be scored as 0 or 1. Certain elements (i.e. decontamination of hospital linens; sub-questions related to building, latrines, waste sorting, and destruction; and alerts) were subsequently recognised as not applicable to level III health facilities, nor could they be achieved in an emergency response, and were therefore removed. The final instrument included 26 elements across 10 sections. The scorecard was implemented monthly in all 65 supported health facilities. The baseline assessment was conducted between 20 April 2020 and 29 April 2020. Data were collected by the health zone IPC supervisor.

#### Triage activities

ACF collected weekly data on the implementation of IPC activities in the supported health facilities. These included the number of persons who went through triage, the number of persons who were isolated (i.e. the number of alerts), the number of alerts investigated by the investigation team, the number of alerts validated by the investigation team, and the number of alerts validated within 24 h.

#### Routine health services

We extracted monthly data on service delivery of eight essential services from the National Health Information System: the total number of monthly outpatient consultations; the number of consultations for malaria, diarrhoea, and acute respiratory infections (ARI); the first antenatal visit (ANC1), fourth antenatal visit (ANC4), and number of institutional deliveries; and the number of children under 12 months of age vaccinated against measles. The pre-COVID period expands from 1 January 2019 to 31 March 2020, and the COVID-19 period from 1 April 2020 to 30 November 2020.

For months where the number of consultations is reported as 0, but there is a non-zero number of consultations for malaria, ARI, or diarrhoea, we replace the number of consultations as missing.

We include health facilities with a completeness of reporting of at least 75% for the indicator of interest and at least 50% of non-zero values. ARI and diarrhoea were excluded from the analysis for Gombe because of high levels of missing data.

### Analytical approach

#### Triage implementation and IPC scorecard

The implementation of triage activities was investigated via descriptive statistics and evolution over time. The IPC capacity of health facilities (overall score and by section) was assessed visually monthly. Factors associated with increased triage capacity were investigated with a mixed-effects model as follows:

$$y_{ij}=NB\left(y_{ij}|\mu_{ij},\theta \right)$$

$$log(\mu_{ij})=\alpha_{i}+\beta_{1}week_{ij}+\beta_{2}consultations_{ij}+\beta_{3}vaccinations_{ij}+\beta_{4}ANC1_{ij}+\beta_{4}ANC1_{ij}+\beta_{6}deliveries_{ij}+\gamma_{m}IPCscore_{mij}$$

where $y_{ij}$ is the outcome of interest (number of individuals triaged), $\alpha_{i}$ is the random intercept (variable for each health facility *i*), *week* is the week number at time *j*, *consultations* is the number of outpatient consultations in week *j* in health facility *i*, *vaccinations* is the number of individuals vaccinated in week *j* in health facility *i*, *ANC1* and *ANC4* are the number of first and fourth antenatal consultations in week *j* in health facility *i*, *deliveries* is the number of deliveries in week *j* in health facility *I*, and *IPC score* is the overall IPC score in week *j* in health facility *i*. NB denotes a negative binomial model, and we allow for first-order autocorrelation. We carry out the analysis in each of the three health zones separately.

For isolation, we use the following model:

$$y_{ij}=NB(y_{ij}|\mu_{ij},\theta )$$

$$log(\mu_{ij})=\alpha_{i}+\beta_{1}week_{ij}+\beta_{2}triaged_{ij}+\gamma_{m}IPCscore_{mij}$$

where *triaged* is the number of individuals triaged in week *j* in facility *i*.

#### Health service utilisation

We used a mixed model with random intercept and long-term trend varying for each health facility. COVID-19 effects are assumed to be the same for all health facilities within the same health zone but can vary across health zones. We use the following model:

$$y_{ij}=NB(y_{ij}|\mu_{ij},\theta )$$

$$log(\mu_{ij})=\alpha_{0i}+\alpha_{1i}month+\beta_{1}COVID\_period+\beta_{2}COVID\_month+\beta_{3}season_{i}+\beta_{4}pneumonia_{i}$$

where $y_{ij}$ is the number of consultations at health facility *i* in month *j*; NB denotes negative binomial function; $\alpha_{0i}$ is the facility-specific intercept; $\alpha_{1i}$ is the facility-specific coefficient for long-term trend; *month* is the variable for month of study, centred at the beginning of the COVID-19 period; COVID_period is a variable taking value 0 in the pre-COVID-19 period (January 2019 to March 2020) and value of 1 in April 2020 onwards; *COVID_month* is the month since the beginning of COVID-19 period; *season*_*i*_ is a dummy variable for 11 months of the year (excluding January) and *pneumonia*_*i*_ is a dummy variable for the period with pneumonia outbreak (December 2019 to February 2020).

As sensitivity analysis, we considered different ways to include seasonality (taking out the *season*_*i*_ terms or fitting cubic splines) and using a quasi-Poisson model instead of negative binomial. For the results of these analyses, please see Supplementary material.

For Gombe, because there had been a lockdown period from 6 April 2020 to 29 June 2020, we included an additional term for the lockdown and post-lockdown period. That is,

$$log(\mu_{ij})=\alpha_{0i}+\alpha_{1i}month+\beta_{1}lockdown\_period+\beta_{2}lockdown\_month+\beta_{3}season_{i}+\beta_{4}pneumonia_{i}+\beta_{5}postlockdown\_period+\beta_{6}postlockdown\_month$$

The *pneumonia* dummy variable was only included in the analysis of overall consultations and ARI. For vaccination, ANC1, ANC4, and deliveries, we used a model without seasonal dummy terms.

We report parameter estimates using the incidence rate ratio (IRR) and related 95% confidence intervals (CIs). For each outcome, we present the level change at the beginning of the COVID-19 period and the trend change for the COVID-19 period. For Gombe, results are disaggregated for the lockdown and the post-lockdown period.

We also calculate two measures of the difference with expected values: (1) the cumulative difference between the observed and expected number of consultations (by type) over the study period and (2) the average monthly percent change in consultations for each month of the COVID period and at each facility within each health zone. See details in Supplementary material (section 2.1).

All analyses were conducted using R V.4.0.5, with the package *mgcv* [13].

## Results

### Description of the health facilities included in the study

Table S1 in supplementary material summarises the characteristics of the health facilities included in the baseline assessment and those finally included in ACF’s intervention. Most (78%) of the supported health facilities were private, 39% had functioning latrines, 55% had access to a water supply system, one-third had a waste triage system, and one-fifth had an IPC focal point.

### Infection prevention and control

**Figure 2.**
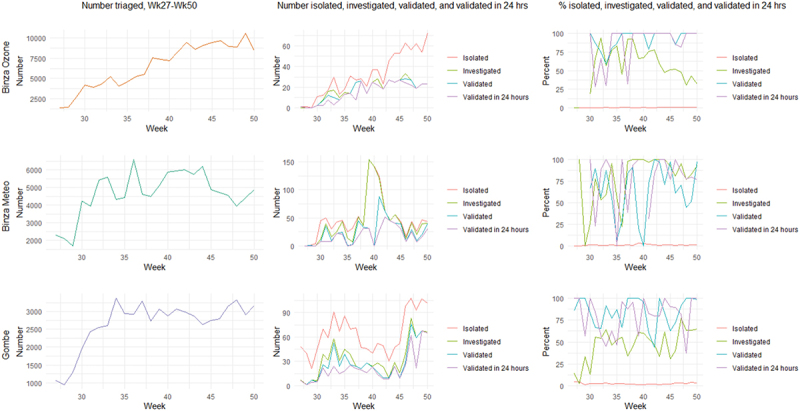
Evolution of key triage parameters over time, by health zone.

Figure 2 shows the evolution of key triage and alert investigation parameters over the study period. The number of triaged people increased in all health zones over time, reflecting the implementation of triage-related activities, such as setting up triage points and providing thermometers and triage forms. The number of isolated persons also increased, but the absolute numbers remained very low over the study period, leading to low proportions in all three zones. The proportion of investigated alerts varied by week but was relatively high in both Binza Meteo and Binza Ozone, where it started declining from October 2020. It remained around 50% in Gombe. Total validated alerts and validated alerts within 24 h also varied substantially by week; absolute numbers were low (i.e. a few dozen per health zone), and the proportion was high and erratic in the three zones.

**Figure 3.**
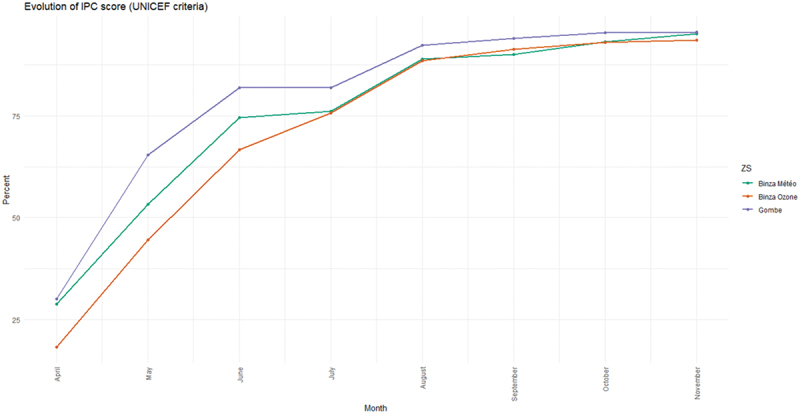
Evolution of IPC score by health zone, from April to December 2020.

**Figure 4.**
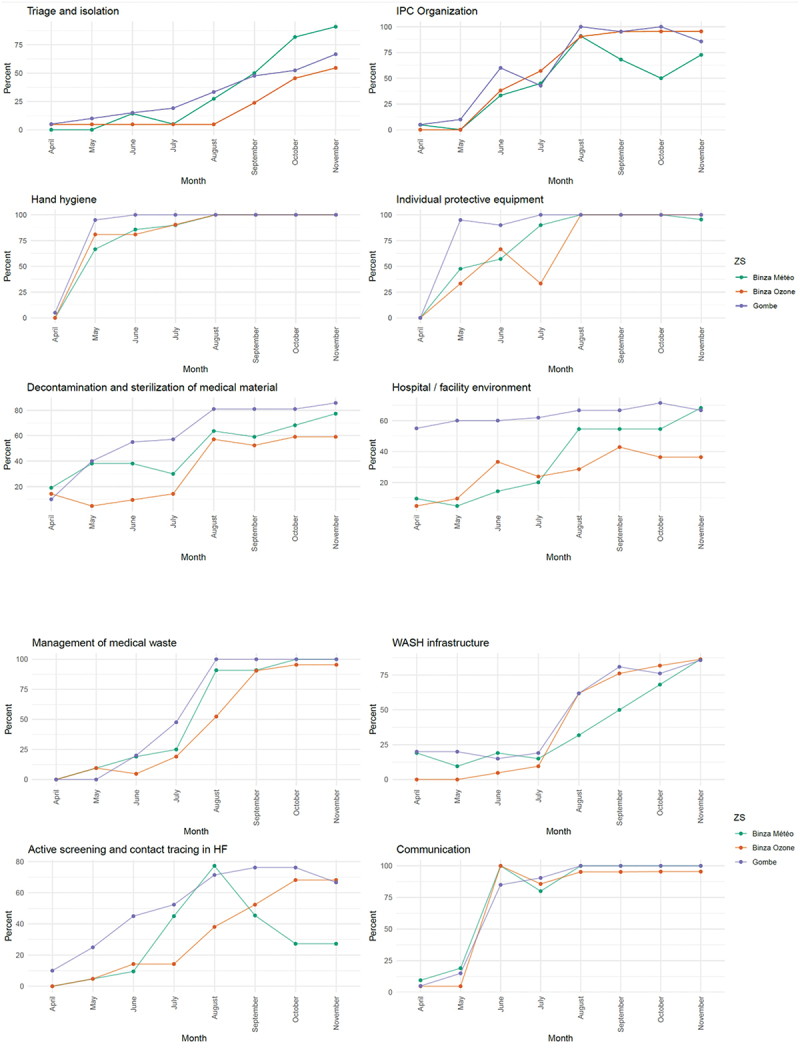
Evolution over time of IPC scorecard subcomponents by health zone (from April to November 2020).

Figures 3 and 4 show the evolution of the total IPC score and by component over the programme period. The overall score improved over time, with all health zones reaching an average score higher than 90% by the end of the programme. Improvements by scorecard component followed different dynamics, with gradual but steady increases in triage and isolation capacity, IPC organisation, health facility environment, and active screening and case search. Other components, such as hand hygiene, PPE, medical waste management and wash infrastructure, experienced more abrupt improvements, likely due to the sudden availability of key equipment or the construction of infrastructure.

Table 1 summarises the regression results and shows that the IPC score was positively associated with increased triage capacity in Binza Ozone and Gombe (6% and 5% increases in triage capacity, respectively, a one-point increase for each in the IPC score) but not in Binza Meteo. The IPC score was also positively associated with isolation capacity in the same zones (21% increase in Binza Ozone and 10% in Gombe).

**Table 1.** Factors associated with increased IPC capacity in health facilities by health zone, Kinshasa, DRC.

	Binza Ozone		Binza Meteo		Gombe	
	IRR [95% CI]	p-value	IRR [95% CI]	p-value	IRR [95% CI]	p-value
*Triage*^a^						
IPC score	**1.06** **[1.03–1.10]**	**<0.001**	1.00 [0.97–1.02]	0.762	**1.05** **[1.01–1.09]**	**0.023**
week	**1.03** **[1.01–1.05]**	**0.003**	**1.04** **[1.02–1.05]**	**<0.001**	1.00 [0.99–1.02]	0.705
*Isolation*						
IPC score	**1.21** **[1.13–1.30]**	**<0.001**	1.02 [0.95–1.08]	0.645	**1.10** **[1.01–1.19]**	**0.021**
week	**1.05** **[1.01–1.09]**	**0.010**	**1.05** **[1.01–1.10]**	**0.025**	1.00 [0.97–1.03]	0.945
Number triaged	**1.001** **[1.00–1.00]**	**<0.001**	**1.002** **[1.00–1.00]**	**<0.001**	**1.004** **[1.00–1.01]**	**<0.001**

^a)^Adjusted by number of consultations, number of vaccinations, ANC1, ANC4, and deliveries.

Bold values are statistically significant at 0.05 level.

### Health care utilisation

Table S2 in supplementary material shows the number of health facilities included in the analysis by indicator and health zone. Results of the interrupted time series analysis are presented in Table 2, and cumulative differences and monthly percent differences are shown in Table 3. Sensitivity analysis is in section 3.3 of the Supplementary material.

**Table 2.** Interrupted time series results for outcome of interest, by health zone, Kinshasa.

	IRR	Overall consultations	ARI consultations	Diarrhoea consultations	Malaria consultations	Vaccinations	ANC1	ANC4	Deliveries
Binza Ozone	Immediate change	0.86 [0.53–1.40]	0.56 [0.28–1.14]	0.89 [0.52–1.52]	0.73 [0.45–1.17]	0.76 [0.33–1.73]	0.96 [0.57–1.61]	1.23 [0.71–2.11]	0.91 [0.56–1.48]
	Immediate change p-value	0.551	0.108	0.660	0.190	0.513	0.884	0.465	0.704
	Trend change	**0.85** **[0.76–0.95]**	1.02 [0.87–1.21]	0.99 [0.87–1.12]	**0.84** **[0.75–0.94]**	0.98 [0.82–1.16]	1.03 [0.93–1.15]	1.01 [0.90–1.13]	0.97 [0.87–1.08]
	Trend change p-value	**0.005**	0.776	0.848	**0.003**	0.773	0.541	0.914	0.591
Binza Meteo	Immediate change	**0.67** **[0.48–0.95]**	0.65 [0.17–2.49]	0.56 [0.27–1.14]	**0.62** **[0.44–0.88]**	0.77 [0.52–1.14]	0.90 [0.65–1.24]	0.85 [0.54–1.34]	0.89 [0.65–1.24]
	Immediate change p-value	**0.023**	0.533	0.108	**0.007**	0.194	0.515	0.485	0.497
	Trend change	1.02 [0.95–1.10]	**1.33** **[1.08–1.64]**	**1.16** **[1.01–1.33]**	1.01 [0.93–1.09]	**1.13** **[1.04–1.23]**	1.01 [0.94–1.08]	**1.19** **[1.09–1.30]**	1.01 [0.95–1.09]
	Trend change p-value	0.584	**0.008**	**0.031**	0.866	**0.003**	0.745	**<0.001**	0.695
Combined	Immediate change	**0.67** **[0.48–0.95]**	**0.48** **[0.28–0.87]**	0.70 [0.46–1.07]	**0.60** **[0.43–0.84]**	0.75 [0.51–1.12]	0.88 [0.67–1.17]	0.83 [0.59–1.15]	0.89 [0.67–1.17]
	Immediate change p-value	**0.023**	**0.016**	0.099	**0.003**	0.158	0.385	0.258	0.393
	Trend change	**0.93** **[0.86–0.99]**	1.12 [0.97–1.27]	1.07 [0.98–1.17]	**0.92** **[0.85–0.98]**	1.08 [0.99–1.17]	1.02 [0.96–1.08]	**1.10** **[1.03–1.18]**	1.00 [0.94–1.06]
	Trend change p-value	**0.032**	0.117	0.141	**0.015**	0.069	0.596	**0.006**	0.989
Gombe	Lockdown immediate change	**0.29** **[0.16–0.53]**	NA	NA	**0.33** **[0.18–0.58]**	**0.27** **[0.10–0.71]**	**0.42** **[0.21–0.85]**	**0.41** **[0.17–1.00]**	1.56 [0.87–2.79]
	Immediate change p-value	**<0.001**			**<0.001**	**0.008**	**0.016**	**0.049**	0.135
	Lockdown trend change	1.02 [0.74–1.39]			1.02 [0.75–1.39]	0.86 [0.51–1.43]	1.15 [0.81–1.63]	1.38 [0.91–2.08]	0.74 [0.54–1.01]
	Trend change p-value	0.912			0.900	0.551	0.448	0.127	0.059
	Post-lock down immediate change	1.16 [0.19–7.13]			0.67 [0.11–4.03]	4.58 [0.33–64.19]	1.11 [0.16–7.60]	5.23 [0.76–35.91]	0.27 [0.04–1.99]
	Post-lock down immediate change p-value	0.874			0.659	0.259	0.919	0.093	0.197
	Post-lock down trend change	1.02 [0.66–1.58]			1.09 [0.71–1.69]	1.10 [0.56–2.16]	0.98 [0.61–1.58]	0.69 [0.41–1.15]	1.36 [0.85–2.17]
	Post-lock down trend change p-value	0.416			0.682	0.783	0.943	0.154	0.195

Bold values are statistically significant at 0.05 level.

**Table 3.** Differences between expected and observed consultations: cumulative difference and average monthly percent change by service and health zone, Kinshasa, April–December 2020.

	IRR	Overall consultations	ARI consultations	Diarrhoea consultations	Malaria consultations	Vaccinations	ANC1	ANC4	Deliveries
Binza Ozone	Cumulative difference	−13,737 [−14,821; −12,697]	−380 [−467; −293]	−72 [−98; −49]	−10,921 [−11,625; −10,192]	−714 [−819; −610]	109 [72; 147]	149 [120; 177]	149 [120; 177]
	Average monthly percent change	−47% [−49%; −45%]	−42% [−47%; −37%]	−15% [−19%; −10%]	−56% [−58%; −55%]	−28% [−31%; −25%]	10% [6%; 14%]	25% [20%; 32%]	25% [20%; 32%]
Binza Meteo	Cumulative difference	−4,835 [−5,260; −4,414]	159 [137; 181]	−20 [−38; 0]	−4,143 [−4,481; −3,825]	365 [325; 406]	−152 [−177; −126]	423 [401; 444]	−84 [−102; −65]
	Average monthly percent change	−28% [−30%; −26%]	104% [74%; 146%]	−3% [−8%; 4%]	−38% [−40%; −36%]	15% [13%; 18%]	−7% [−9%; −6%]	66% [60%; 73%]	−6% [−7%; −4%]
Combined	Cumulative difference	−25,981 [−27,088; −24,972]	−300 [−347; −250]	−97 [−118; −75]	−18,897 [−19.634; −18,228]	−500 [−591; −414]	−215 [−243; −187]	259 [242; 276]	−315 [−337; −294]
	Average monthly percent change	−48% [−49%; −47%]	−27% [−30; −24]	−11% [−13%; −9%]	−55% [−56%; −54%]	−10% [−12%; −8%]	−6% [−7%; −6%]	16% [15%; 18%]	−12% [−13%; −11%]
Gombe	Cumulative difference	−36,091 [−38,525; −33,897]	NA	NA	−22,032 [−23,311; −20,576]	−839 [−1,086; −588]	−131 [−145; −117]	7 [3; 12]	−44 [−48; −39]
	Average monthly percent change	−60% [−61%; −58%]			−63% [−64%; −61%]	−20% [−25%; −15%]	−29% [−31%; −27%]	10% [3%; 18%]	−19% [−21%; −17%]

**Figure 5.**
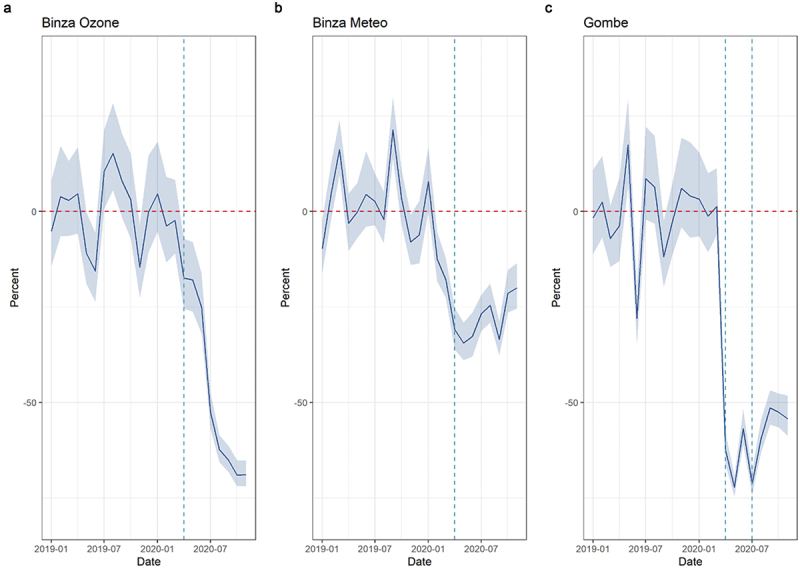
Average monthly percent difference in expected and observed outpatient consultations in Kinshasa, by health zone, from January 2019 to November 2020. Dashed vertical lines represent the beginning of the COVID-19 period (1 April 2020); for Gombe health zone only, the second dashed line (1 July 2020) represents the end of the 3-month-long lockdown period.

Overall consultations showed a decrease at the beginning of the COVID-19 period in all zones: by 33% in Binza Meteo and Binza Ozone combined (IRR: 0.67, 95%CI [0.48–0.95]) and by 71% in Gombe (IRR: 0.29, 95%CI [0.16–0.53]) at the beginning of the lockdown. Consultations increased again during the lockdown and continued to do so after the lockdown was lifted. No major increase can be seen after the lockdown in Gombe. These decreases corresponded to a total of 25,981 fewer consultations in Binza Meteo and Binza Ozone combined (a 48% average monthly decrease) and to 36,091 missed consultations in Gombe alone (60% average monthly decrease) (Figure 5).

**Figure 6.**
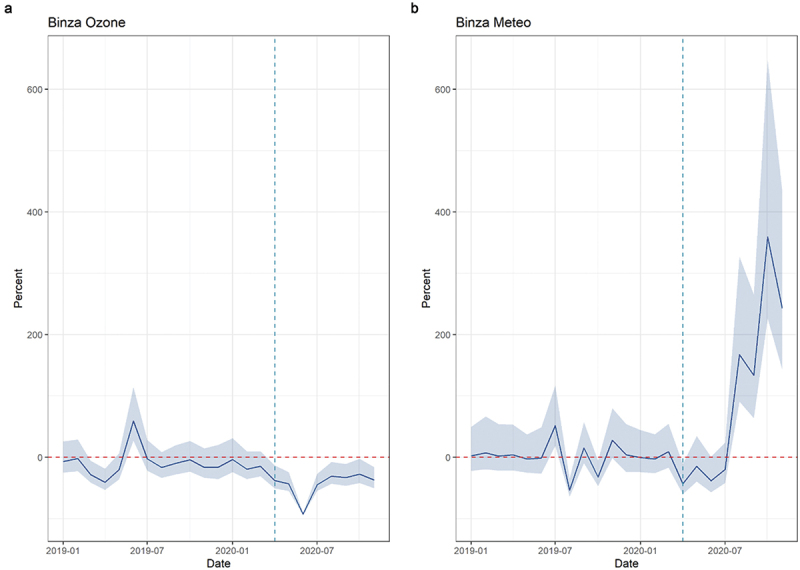
Average monthly percent difference in consultations for respiratory tract infections, Kinshasa, by health zone, from January 2019 to November 2020. Dashed vertical lines represent the beginning of the COVID-19 period (1 April 2020).

**Figure 7.**
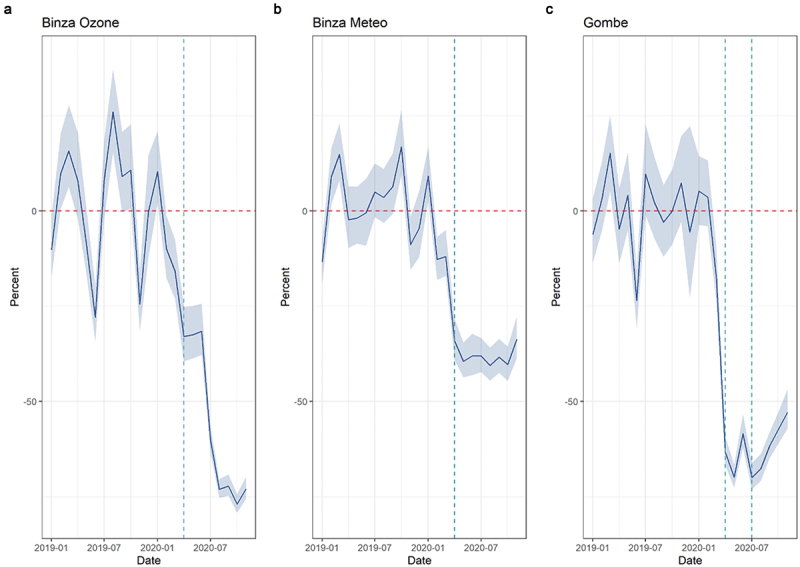
Average monthly percent difference in consultations for malaria in Kinshasa, by health zone, from January 2019 to November 2020. Dashed vertical lines represent the beginning of COVID period (1 April 2020); for Gombe health zone only, the second dashed line (1 July 2020) represents the end of the 3-month-long lockdown period.

Regarding consultations for infectious diseases, ARI consultations reported an immediate decrease by 52% in Binza Ozone and Binza Meteo combined (IRR: 0.48, 95% [0.28–0.87]). Some catching up was seen in Binza Meteo during the COVID-19 period (change in slope IRR: 1.33, 95%CI [1.08–1.64]). This decrease corresponded to 300 fewer consultations in the two zones combined and a 27% average monthly decrease (Figure 6). Diarrhoea consultations showed erratic patterns in the pre-COVID-19 period and were few on average. Immediate drops can be seen in both zones, but the results were not statistically significant. Malaria consultations showed a decrease in all zones: by 40% in Binza Meteo and Binza Ozone combined (IRR: 0.60, 95%CI [0.43–0.84]) and by 67% in Gombe (IRR: 0.33, 95%CI [0.18–0.58]) at the beginning of the lockdown. Little to no catching up was observed in trends. These reductions corresponded to 18,897 fewer consultations for Malaria in Binza Meteo and Binza Ozone combined and to 22,032 fewer consultations in Gombe (the average monthly change was 55% and 63%, respectively) (Figure 7).

Vaccination decreased in all zones, but results are statistically significant only in Gombe, where a 73% drop was recorded (IRR: 0.27, 95%CI [0.10–0.71]). An increase is seen in the post-lockdown period, but the results are not statistically significant (CIs are large as few health facilities met inclusion criteria). The average monthly difference in Gombe was estimated at −20%.

Maternal health services seem to have been little affected in Binza Meteo and Binza Ozone. While decreases were seen at the beginning of the COVID-19 period, results were not different from pre-COVID-19 trends for ANC1 and ANC4. The trend over time for ANC4 was positive, reflected in 259 more consultations for the two zones combined. On the contrary, Gombe reported a 58% (IRR: 0.42, 95%CI [0.21–0.85]) and 59% drop (IRR: 0.41, 95%CI [0.17–1.00]) for ANC1 and ANC4, respectively, at the beginning of the COVID-19 period. Trends over time and post-lockdown showed some minor catching up. Institutional deliveries seemed little affected in the three health zones.

## Discussion

This study reports on the implementation of IPC interventions in health facilities in Kinshasa, DRC, during the first 9 months of the COVID-19 pandemic. It also investigates how health services utilisation changed over the same period.

The goal of IPC measures in health facilities is to prevent health care–associated infections, i.e. infections that could occur while providing or receiving care in health facilities so that health services are provided safely for patients, health care providers, and visitors [14]. Given the high transmissibility of SARS-CoV-2 and the little understanding of the virus’ behaviour at the pandemic’s beginning, the MoH and partners quickly understood the importance of increasing IPC measures in a city like Kinshasa. Previous experience during other epidemics showed the limited preparedness of health facilities to respond to outbreaks in the DRC. For example, during the 2018–2020 Ebola in North Kivu and Ituri, a large proportion of the Ebola cases (20%) were nosocomial [15]. In the most recent large cholera outbreaks in Kinshasa, health facilities exhibited a low level of cholera preparedness despite previous experience [16]. IPC scores at baseline were low, likely due to limited financial and human capacity to implement IPC. Most of the health facilities that were supported in Kinshasa are private, tend to be small, and have high staff turnover. IPC is rarely a priority for private facilities, as they often lack the financial capacity to implement and sustain IPC measures. Therefore, the support of an external partner and monthly supervision visits were necessary to increase IPC standards. Unfortunately, the time-bound support casts doubt on the sustainability of the measures. While infrastructural improvements that facilitated access to water and waste management could likely continue after the programme’s end, the procurement of PPE was more challenging. Locally manufactured solutions, such as alcohol-based hand rub, were introduced in other countries [17] and contributed to sustaining improved hand hygiene. Future efforts in this direction could increase response capacity in the DRC too.

IPC support entailed several components that faced different implementation challenges. This was reflected in the distinct evolution dynamics of the IPC score. Some components (hand hygiene, PPE availability, and communication) improved promptly as the setting up of hand washing stations and distribution of PPE occurred quickly. Communication activities encompassed dialogues with community leaders to increase community support and improved information sharing, mainly by health workers at health facilities and community health workers. For other components, improvements took longer. For example, setting up and implementing triage and isolation needed 5–6 months to reach 50%. Similarly, medical waste management achieved satisfactory levels for the three zones only in September, likely because of procurement delays and construction time. Active screening and contact tracing improved slowly between April and August and then plateaued in Binza Ozone and Gombe. In Binza Meteo, the score started decreasing after August. Informal reports of threats to health care workers after isolating patients and notifying alerts began at that time, which may have disrupted the implementation of isolation activities in Binza Meteo.

When looking at the continuum from triage through case management, triage capacity improved, reflecting the increasing number of people whose temperatures were taken at the facility entrance. Yet, isolation and investigation capacity did not improve at the same pace. Enforcing isolation was challenging due to the reluctance of patients to be isolated (as previously seen during Ebola outbreaks [18]) and to the limited isolation space available in health facilities. Facilities in these health zones often occupy small buildings, and securing the physical space to set up isolation areas in compliance with IPC guidelines was not always feasible or easy.

The next bottleneck in the continuum was at the investigation step. In the first months of the pandemic (April to mid-September 2020), investigation teams were external to the health zone office. When a patient met the suspect case definition, the health facility notified the health zone office, which in turn notified the surveillance team of the national surveillance commission. The surveillance team was tasked to investigate the alert. Due to the limited number of investigation teams, insufficient transportation means, and the high number of alerts, the investigation teams could not promptly conduct each investigation; instead, patients had to wait, sometimes for hours. Some patients ended up leaving the health facility before the investigation team arrived, contributing to the low proportion of alerts that were investigated. From mid-September 2020, investigation responsibility was decentralised and transferred to the health zone office, which had to train health facility staff to conduct the investigation. While this second approach reduced waiting time, it also diminished the independence of the investigation team, as it was now composed of health facility workers who knew the patients and were subject to possible pressures. Fears to be confronted or defamed/labelled as a facility where it was more likely to be investigated for COVID-19 were reported and likely influenced the health care workers’ attitude towards the investigation. Furthermore, notifying an alert meant referring a patient, as COVID-19 cases were treated in specific facilities. This, in turn, meant a loss of income for the facility. Health care workers in private health facilities may feel more accountable to the owner/manager of the facility rather than to the government, as they do not receive public support, and their only income is service fees. Integrating private health facilities into the national system remains a priority for the DRC’s government to ensure compliance with standards and the overall quality of health care [19].

The above challenges raise questions about operationalising a generic clinical case definition in low-resource settings where existing capacity could not handle the number of suspected cases. Given how generic COVID-19 symptoms are, access to testing and a quick result turn-around to rule out COVID-19 promptly would have been critical for effective validation of alerts. Ensuring sufficient testing capacity remains one of the key components in future epidemic responses [20,21].

Health service utilisation experienced fluctuations during the study period. All types of services we investigated (general consultations, consultations for infectious diseases and preventative services) experienced a decrease in Gombe, where the lockdown was implemented. Movement exemptions for patients, as implemented in other countries, were foreseen in Kinshasa, too. However, blockages and delays served as major deterrents, and many people likely preferred not to attempt the journey to the health facility. Results from Binza Meteo and Binza Ozone, where no lockdown was enforced, also point to decreases in utilisation of several services, but some are not statistically significant. This is likely due to a lack of power, as the number of health facilities included in the analysis is limited (especially for certain indicators for which data were missing). Preventative maternal and child health services were less affected in the health zones without lockdown. Our results are in line with the other study from Kinshasa [9] that covered a similar period and with results at the national level [6], other low- and middle-income countries, and humanitarian contexts [7,22–24].

Several factors likely worked as a deterrent to seeking health care, including the fear of being infected, the triage and isolation process, the fear of being tested, the time needed for the investigation, and the risk of being transferred to COVID-19 facilities. This was likely particularly relevant for malaria and other respiratory infections with symptoms like COVID-19. Delays in obtaining test results also caused important disruptions to suspect cases’ lives, possibly reducing their willingness to be tested. Until May 2020, the only testing capacity for the entire country was in Kinshasa and included five PCR (Polymerase chain reaction) machines. Such limited capacity led to sample wastes, delays between sample collection and testing, and delays in sharing results. From August 2020, rapid diagnostic tests were introduced; however, availability remained low. The fear of ‘indefinite forced quarantine’ has been reported in other studies from Kinshasa and Goma [25]. As previous experience from Ebola outbreaks showed, communication with communities to clarify possible misunderstandings and fears related to control measures is essential to ensure patients understand modifications to health services provision and continue seeking care [26].

The social and economic consequences of lockdown, border closure, and a lack of employment cannot be underestimated. About half of the households in Kinshasa reported unemployment or experiencing decreased labour income in June–July 2020 [27]. While this proportion declined over the study period (14% in December 2020), households were forced to adjust their priorities. Given the low level of public funding and the high reliance on user fees to finance health services in the DRC [28], an increase in foregone or delayed health care was to be expected. Introducing free health care during COVID-19, as previously done during the Ebola outbreak in Equateur in 2018 [29], could have helped sustain health care utilisation. Yet, it is difficult to predict how the same intervention (free care) could have influenced individual behaviour in a very different situation, both in terms of the virus (SARS-CoV-2 is nothing like the Ebola virus) and context (a global pandemic versus a localised epidemic). Certainly, the extent of the COVID-19 pandemic goes well beyond the Ebola outbreak that affected only three health areas in one province and was declared over within a few weeks.

This study has some limitations. First, data availability varied by indicator and affected the model fit. Deviations in the pre-COVID-19 period point to difficulties fitting the model and the fact that other factors (besides COVID-19) disrupted expected trends. However, as no systematic bias can be seen in the pre-COVID-19 period, and the changes at the beginning of the COVID-19 period are more remarkable, we consider the results reliable. Second, cumulative estimates of the changes in consultations should be considered as indicative, pointing towards an approximated magnitude. As they are calculated against the counterfactual, they are based only on the health facilities included in the analysis. They are likely an underestimation, as facilities that did not report or that closed completely were not included.

## Conclusion

Despite numerous implementation challenges, IPC capacity improved in the supported health facilities during the programme. The effectiveness of the triage and alert validation process was affected by the complexity of implementing a broad clinical definition in a limited resource setting with a pre-pandemic epidemiological profile characterised by infectious diseases with symptoms similar to COVID-19. Increasing IPC preparedness via training and prepositioning of PPE and ensuring testing capacity remain essential for future epidemic and pandemic response to improve the understanding of the disease and maintain health services. The DRC has a unique capacity to respond to epidemics, and further discussion is needed to adapt response strategies to different pathogens.

## Supplementary Material

Supplemental Material

## Data Availability

Data were obtained from the National Health Information System and should be requested from DRC MoH.

## Paper context

Infection prevention and control activities were a central component in the COVID-19 response and supported triage, alert investigation, and surveillance activities. However, implementing triage and alert validation processes in a low-resource setting with a pre-pandemic epidemiological profile characterised by infectious diseases with symptoms like COVID-19 is challenging, not always feasible, and little documented. Context-specific considerations are required, and testing capacity remains key for future pandemic responses.
