# Supplementary material for: The implementation of infection prevention and control measures and health care utilisation in ACF-supported health facilities during the COVID-19 pandemic in Kinshasa, Democratic Republic of the Congo, 2020
Source: Glob Health Action. 2023 Oct 17;16(1):2258711. doi: 10.1080/16549716.2023.2258711 (PMC10583608; doi:10.1080/16549716.2023.2258711)
Supplement: Supplemental Material [file ZGHA_A_2258711_SM8214.docx]

**The implementation of infection prevention and control measures and health care utilization in ACF-supported health facilities during the COVID-19 pandemic in Kinshasa, Democratic Republic of the Congo, 2020**

**Supplementary material**

Table of Contents

[1 Additional information about the methods 1](#_Toc147379011)

[1.1 Difference from expected values 1](#_Toc147379012)

[2 Additional results 2](#_Toc147379013)

[2.1 Characteristics of health facilities included in ACF program 2](#_Toc147379014)

[2.2 Health facilities included in the analysis 3](#_Toc147379015)

[2.3 Sensitivity analysis of utilization of health services 3](#_Toc147379016)

**List of tables**

[Table S1: Characteristics of health facilities supported by ACF and included in the study. 2](#_Toc147379021)

[Table S2: Number of health facilities included in the analysis by outcome of interest and health zone. 3](#_Toc147379022)

# Additional information about the methods

## Difference from expected values

To do so we first generated 1,000 predicted values (“expected”) had there been no changes during the COVID period using the base model, after setting the $COVID\_period$, $COVID\_month$, $lockdown\_period$, and $postlockdown\_month$ terms to 0. If values less than 1 were generated, they were replaced with 1, as we expected at least one consultation at each health facility on a monthly basis. For months where observed data were missing, it was imputed using predicted value from the base model. Difference between observed and expected values at each facility for each month was calculated for the 1,000 draws. For each of the draws, the cumulative difference between observed and expected values was calculated by summing up the difference from each of the health facilities in the health zone. From these 1,000 differences, median, 2.5^th^, and 97.5^th^ quantiles were obtained. To calculate the average monthly percentage difference at health zone level, for each of the 1000 draws, for each month, we calculated the percent difference between cumulative observed and cumulative expected number of consultations for specific service. For each of the draws, we then obtained the average percent difference. The median, 2.5^th^, and 97.5^th^ quantiles were then obtained for the average percent difference.

# Additional results

## Characteristics of health facilities included in ACF program

The total number of health facilities assessed were 83; of which, 65 were finally included in ACF program. Data on type of facility is available for 64 of the supported facilities and results of the IPC assessment for 62 (see inclusion criteria above).

Table S1: Characteristics of health facilities supported by ACF and included in the study.

|  | **All Health Facilities** | **Health Facilities**  **included in the study** |
| --- | --- | --- |
| N of Health facilities assessed | 83 | 65 |
| Type of management |  |  |
| Private | 67% | 78% |
| Religious | 11% | 8% |
| Public | 22% | 16% |
| Type of HF |  |  |
| Health center | 92% | 95%^a^ |
| Hospital | 5% | 0% |
|  |  |  |
| IPC capacity |  | 62 |
| Triage area available | 12% | 11% |
| Isolation area available | 8% | 6% |
| IPC focal point | 19% | 19% |
| HF with IPC management committee | 6% | 6% |
| Functioning hand washing stations | 20% | 16% |
| Monitoring of PPE availability | 14% | 8% |
| PPE stock available at any time | 1% | 2% |
|  |  |  |
| WASH infrastructure |  |  |
| HF with functioning latrines | 41% | 39% |
| HF with access to water supply system | 61% | 55% |
| HF with waste triage system | 37% | 31% |
|  |  |  |
| Scorecard results |  |  |
| Less than 50% | 94% | 95% |
| More than or equal to 50% | 6% | 5% |

*Notes: ^a^ The remaining 5% of health facilities (i.e., 3 health facilities) were either a Health Post (1 facility) or undefined (2)*

## Health facilities included in the analysis

After exclusion of health facilities with 25% or more missing data for each indicator of interest or with all/majority of months reporting 0s, we retained following number of facilities by each health zone

Table S2: Number of health facilities included in the analysis by outcome of interest and health zone.

|  | **Binza Ozone** | **Binza Meteo** | **Gombe** |
| --- | --- | --- | --- |
| N of HF supported by ACF | 22 | 22 | 21 |
|  |  |  |  |
| Data availability > 75% |  |  |  |
| Overall consultations | 17 | 17 | 20 |
| ARI | 11 | 7 | 0 |
| Malaria | 17 | 16 | 19 |
| Diarrhea | 12 | 14 | 0 |
| Vaccination | 7 | 16 | 3 |
| ANC1 | 11 | 17 | 10 |
| ANC4 | 9 | 13 | 3 |
| Deliveries | 11 | 17 | 8 |

## Sensitivity analysis of utilization of health services

| Method |  | Fixed | Random |
| --- | --- | --- | --- |
| lme | lme_simple_fit | month_centered  period2  month_covid  season_i | intercept |
| lme, log(cons) | lme_simple_fit2 | month_centered  period2  month_covid  season_i | intercept |
| glmmPQL; negbinom | m1 | period2  month_covid  season_i | intercept  month_centered |
| glmmPQL; negbinom | m1_period3 | period2  month_covid  period3  period3*month_centered  season_i | intercept  month_centered |
| glmmPQL: quasipoisson, loglink | glmmPQL_fit2 | period2  month_covid  season_i | intercept  month_centered |
| glmmPQL: quasipoisson, loglink | glmmPQL_fit3 | period2  month_covid  period3  period3*month_centered  season_i | intercept  month_centered |
| gam: negbinom | mg | period2  month_covid  season_i | intercept  month_centered |
| gam: negbinom | mg_ran | season_i | intercept  month_centered  period2  month_covid |
| gam: negbinom | mg_pneumo | period2  month_covid  pneumonia  season_i | intercept  month_centered |
| gam: negbinom | mg3 | period2  month_covid  period3  period3*month_centered  season_i | intercept  month_centered |
| gam: negbinom | mg_seas | period2  month_covid  season as cr | intercept  month_centered |
| gam: negbinom | mg_noseas | period2  month_covid  pneumonia | intercept  month_centered |

Binza Ozone: After removing health facilities with 25% or more missing data in consultations, there are 17 facilities that remain.

LME:

Plots seem to provide a reasonable fit for some FOSA, but not for all. For lme_simple_fit, fit is especially poor for CS Libiki, Kerith, La Charite, Le Jourdain, PS Promestra, and St Jean.

For the transformed fit (lme_simple_fit2), it’s especially poor for Kerith, Le Jourdain, and ACC.

It would appear that using a random intercept only model is not a great choice. However, including random slope leads to issues with convergence.


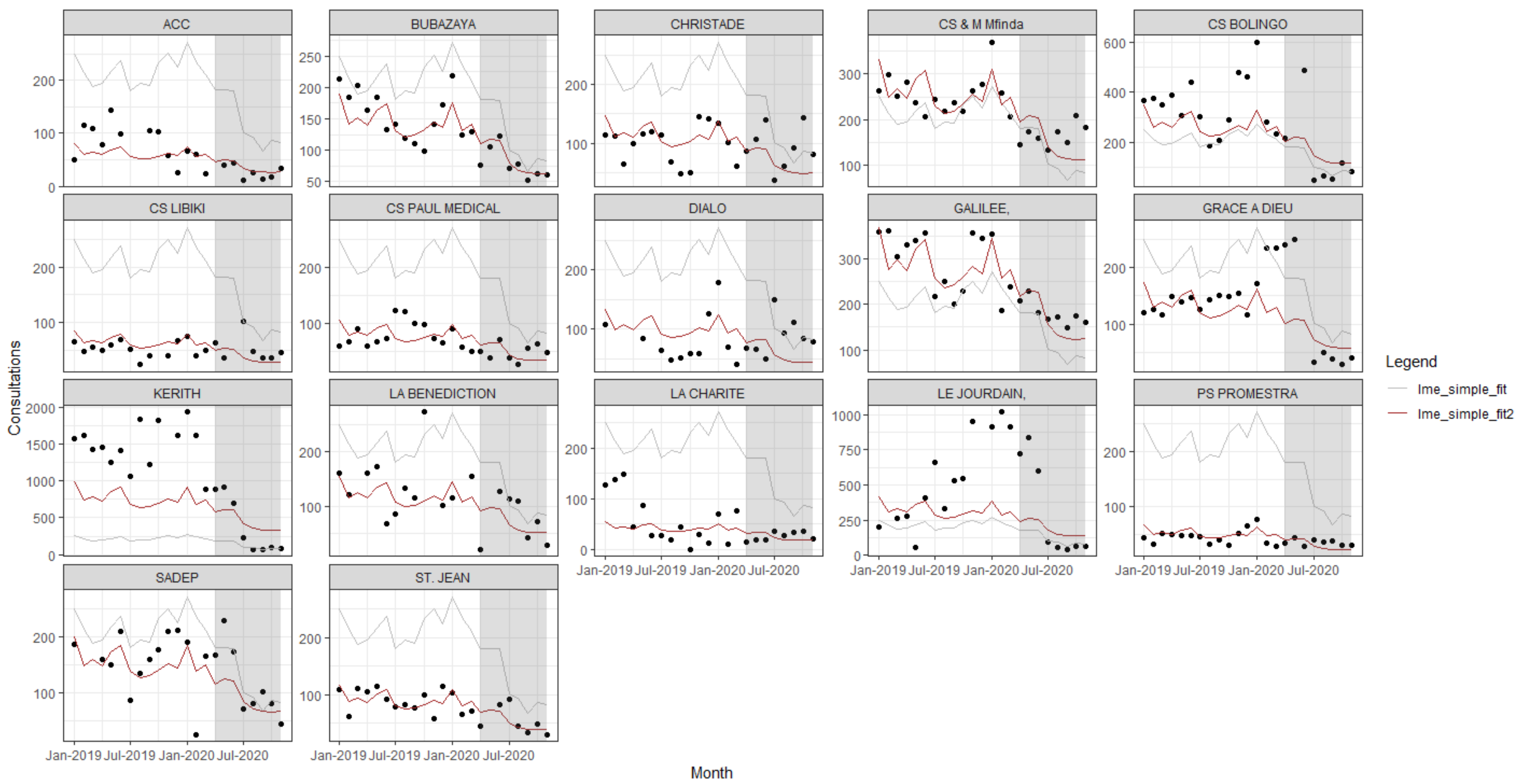


glmmPQL:

Overall, the fit appears to be better than from the lme. Including period3 term does not appear to make much difference, which is consistent because outside of Gombe, there was no lockdown.


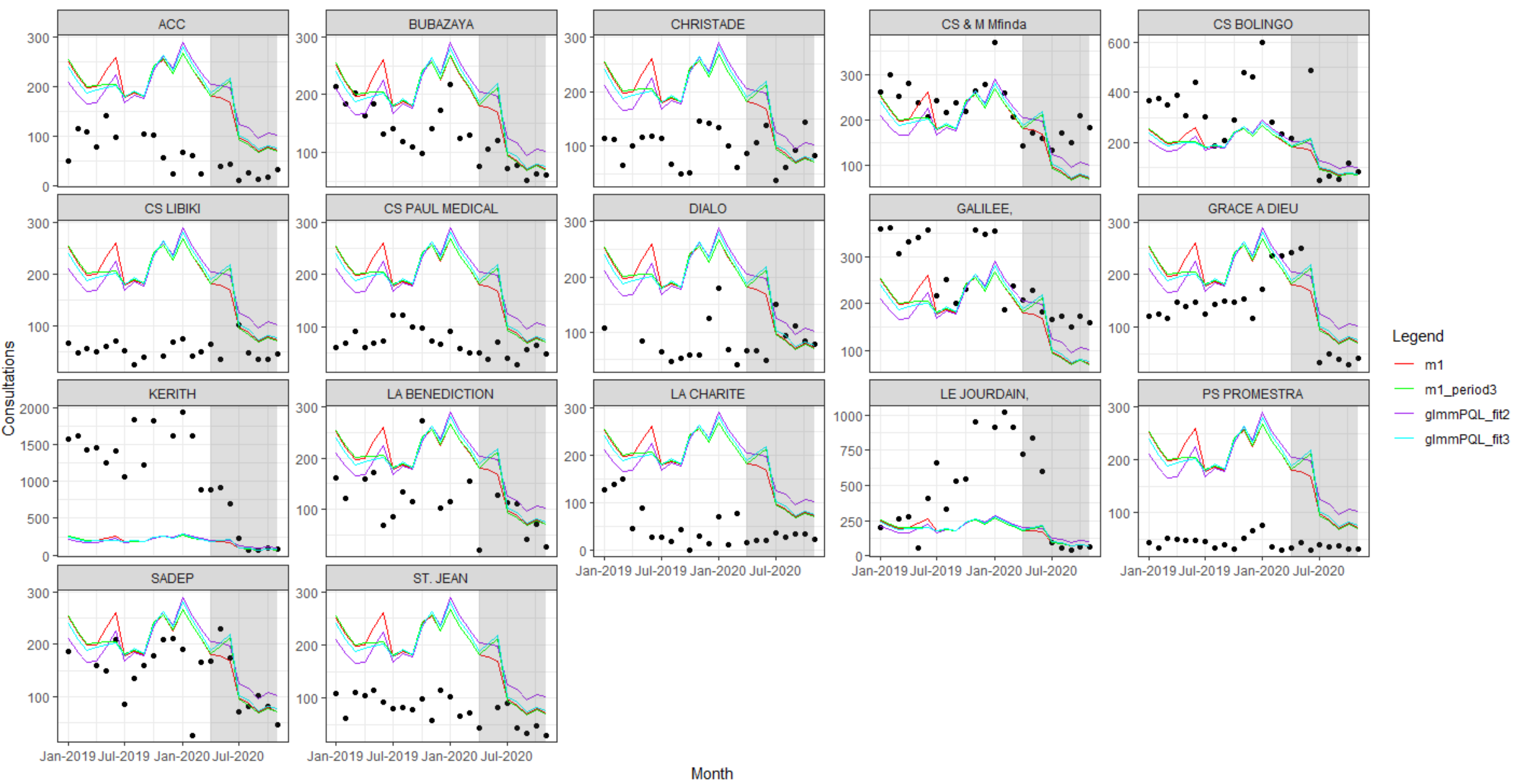


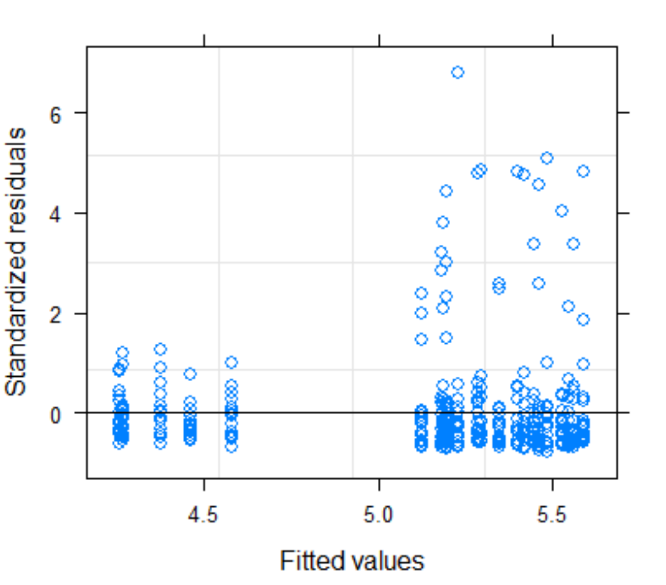
We look at the residuals of glmmPQL_fit2 and for m1.

Figure 1. Residuals for glmmPQL_fit2


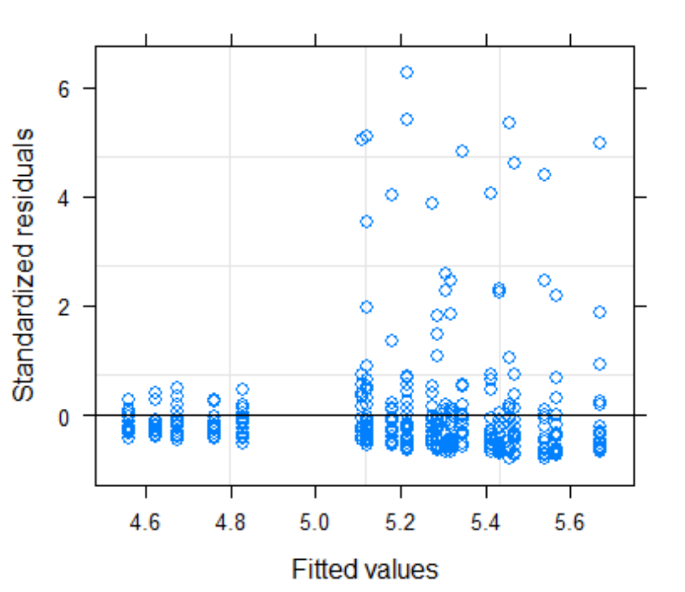


It appears that for glmmPQL_fit2, residuals fan out as fitted values increase, which is problematic. Negative binomial appears better than quasi poisson, so we will use it going forward.

Pearson’s chi squared residuals are below 0.05 for both, which indicates a problem with fit.

Gam
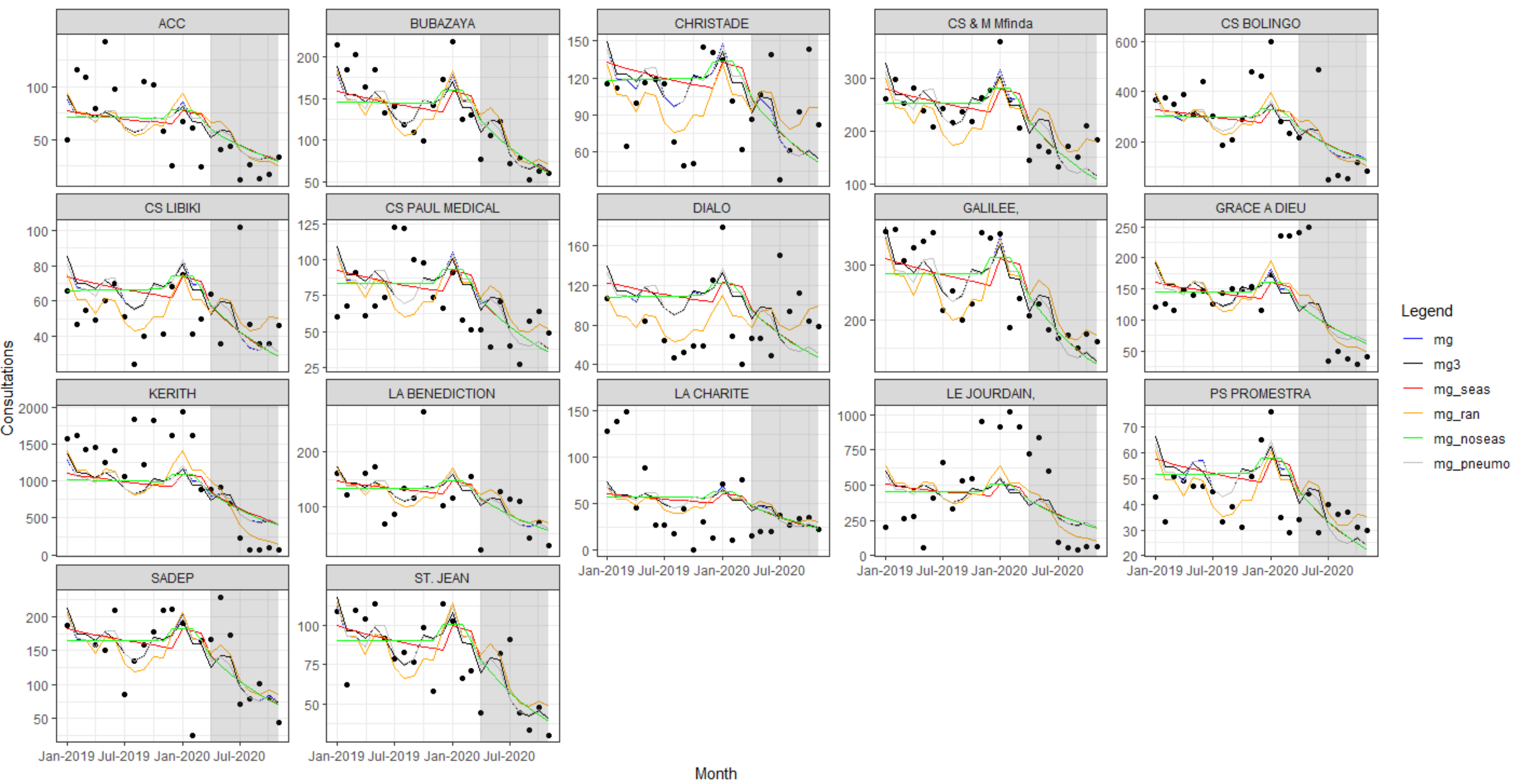


Figure 2. Residuals for m1

Looking at residuals for mg, which is the simplest model, they seem reasonable, and are also generally smaller than for the glmmPQL approach. Furthermore, checking the QQ plots for random effects doesn’t imply a violation of the gaussian assumption.


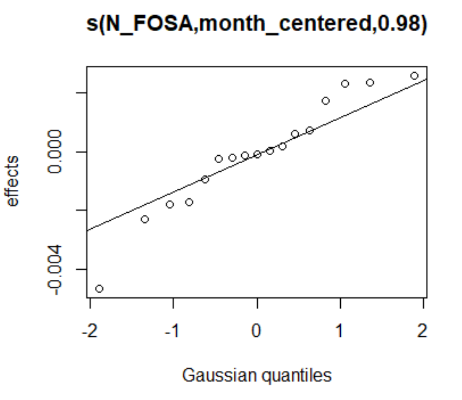

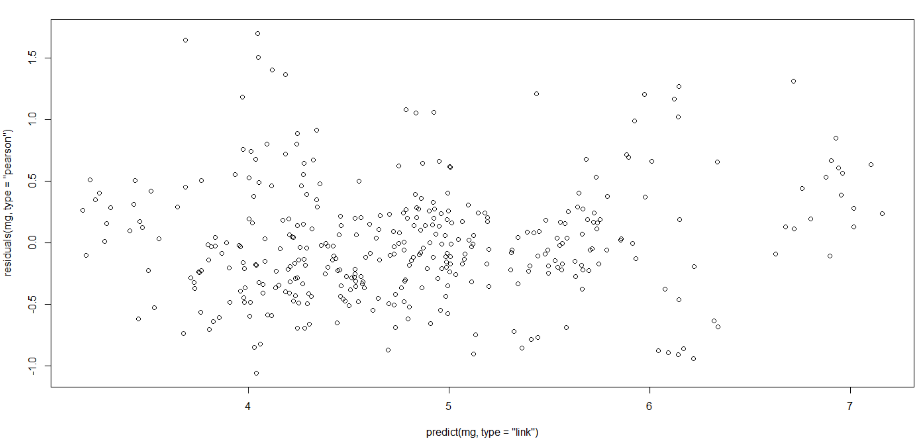


Figure 4. Residuals for mg

Figure 3. QQ plot for random effects

Hence we will move forward with the gam approach.

We compare three models that treat covid period as fixed effect (mg, mg3, mg_seas):

| Model | df | AIC | R^2^ |
| --- | --- | --- | --- |
| mg | 31.81722 | 4322.117 | 0.775 |
| mg3 | 35.23248 | 4325.488 | 0.773 |
| mg_seas | 20.16693 | 4304.243 | 0.767 |
| mg_noseas | 21.06542 | 4309.601 | 0.775 |
| mg_pneumo | 33.11030 | 4323.859 | 0.775 |

The models are somewhat similar; mg_seas has the lowest AIC and the lowest degrees of freedom; however, mg_noseas may be a wise model to use because we don’t have enough time periods to truly fit seasonality.

We will consider the **mg_noseas** and **mg** models going forward. Note that period 3 terms in mg3 are not significant. The results are almost identical.

Meta analysis of effects:

Now, we fit a model independently for each health facility, and pool them together to obtain an estimate of the effects. This meta-analytical method uses inverse variance method and Hartung-Knapp adjustment for random effects model.

Mg no season model (with pneumonia):


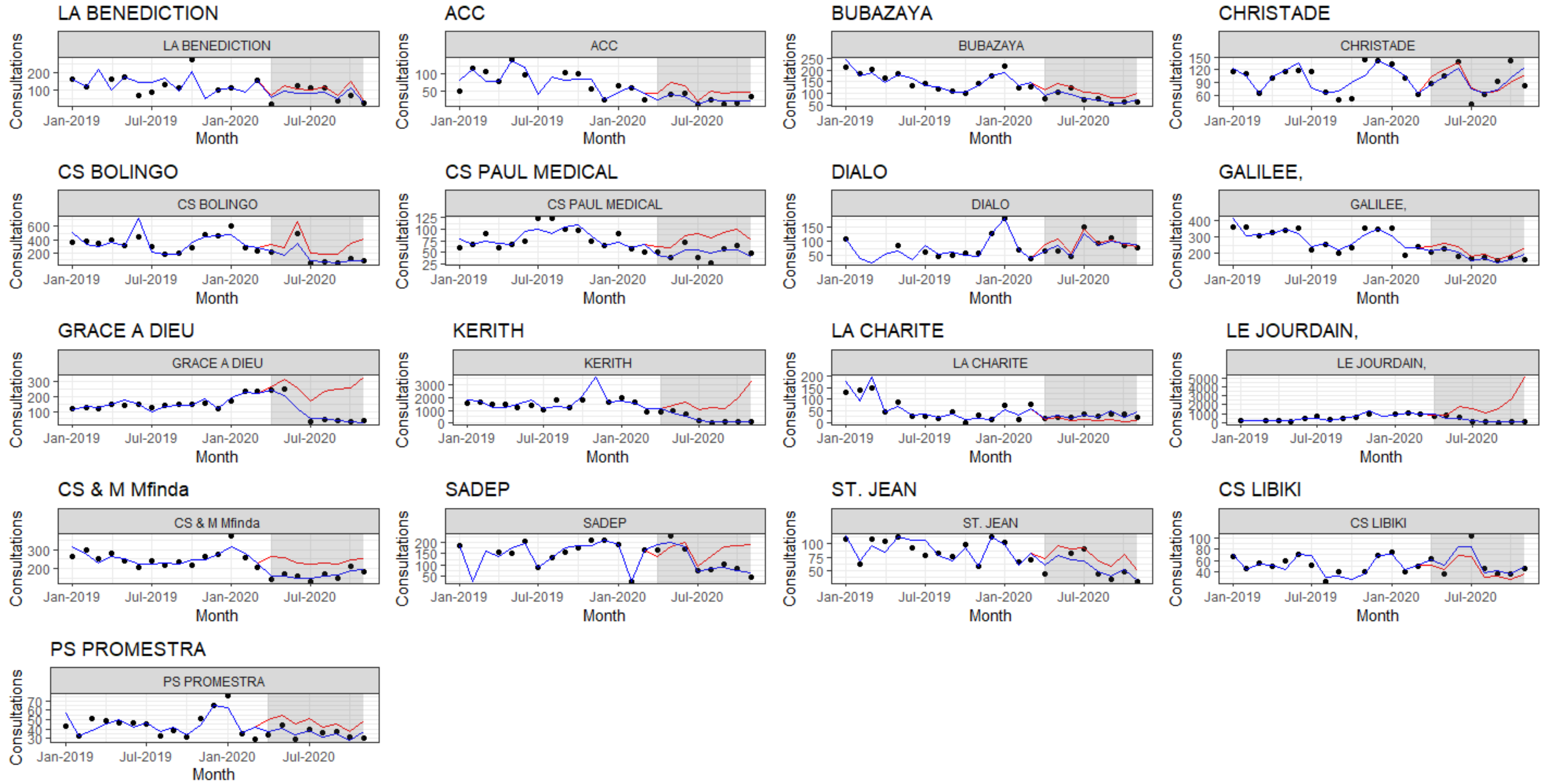


Results

| **Model** | **IRR immediate effect** | **IRR change in slope** |
| --- | --- | --- |
| mg | 0.86 [0.56 – 1.32] | 0.91 [0.82 – 1.01] |
| mg_seas | 0.82 [0.59 – 1.14] | 0.92 [0.85 – 0.99] |
| mg_noseas | 0.86 [0.62 – 1.20] | 0.90 [0.84 – 0.97] |
| mg_pneumo | 0.86 [0.56 – 1.32] | 0.91 [0.82 – 1.01] |
| Meta analysis: mg | 0.87 [0.76 – 0.98] | 0.93 [0.84 – 1.03] |
| Meta analysis: no seasons | 1.07 [0.87 – 1.28] | 0.92 [0.84 – 1.01] |
| Meta analysis: no seasons, yes pneumonia | 0.87 [0.75 – 0.99] | 0.91 [0.83 – 0.99] |

**Binza Meteo**

For Binza Meteo, we only look at mg, mg_noseas, mg_pneumo, and the metaanalysis. Consider mg as the primary model.


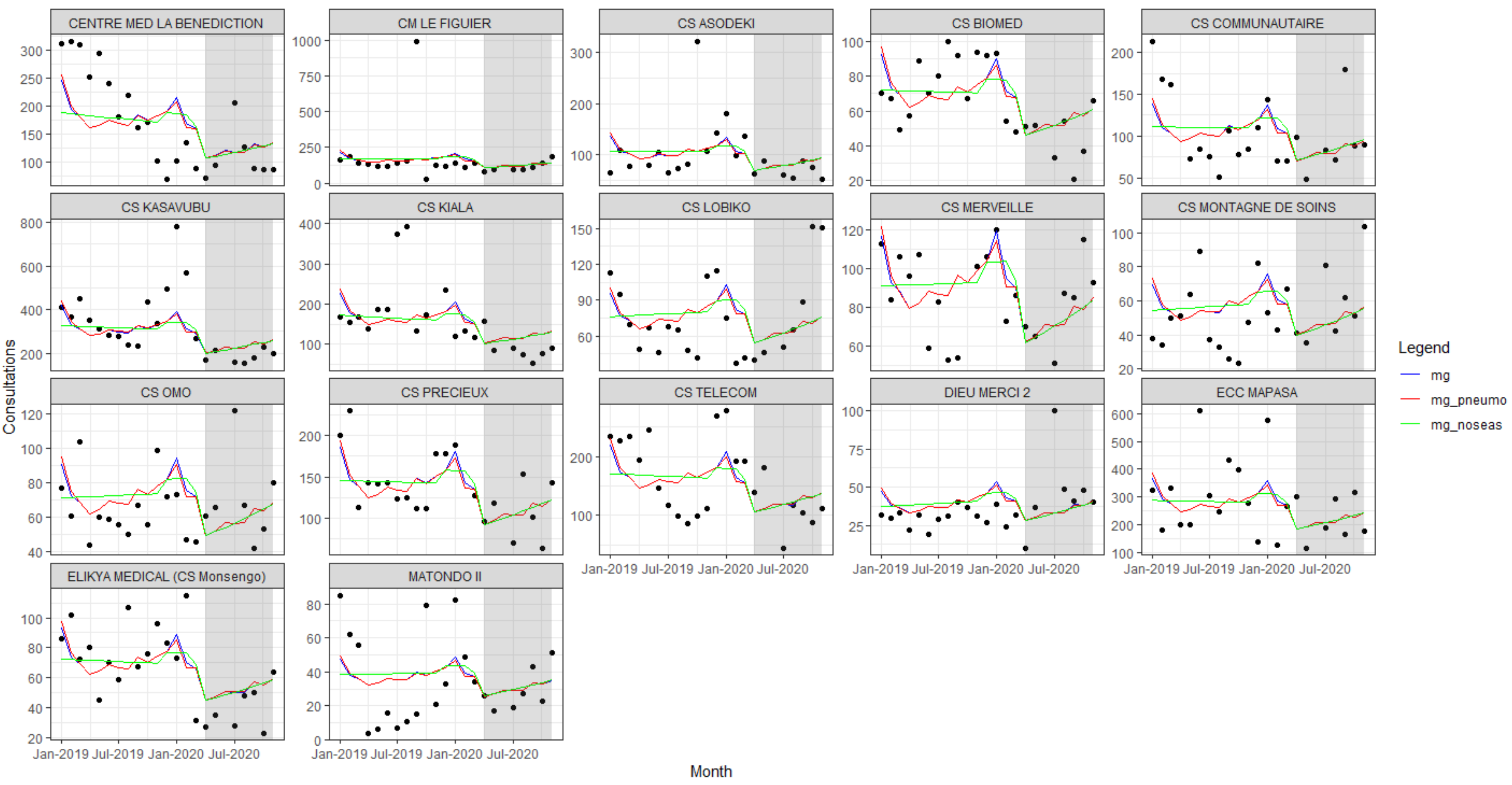


There are only 17 health facilities retained in analysis (with at least 75% of data).

| **Model** | **IRR immediate effect** | **IRR change in slope** | **AIC (df)** | **R2** |
| --- | --- | --- | --- | --- |
| mg | 0.76 [0.54 – 1.09] | 1.01 [0.94 – 1.10] | 3986.816 (35.23) | 0.55386 |
| mg_noseas | 0.66 [0.50 – 0.86] | 1.04 [0.99 – 1.10] | 3974.446 (25.05) | 0.5499 |
| mg_pneumo | 0.76 [0.53 – 1.08] | 1.01 [0.94 – 1.10] | 3987.889 (36.21) | 0.5481 |
| Meta analysis: mg | 1.14 [0.57 – 1.73] | 1.01 [0.95 – 1.08] |  |  |
| Meta analysis: mg + pneumonia | 1.39 [0.62 – 2.15] | 1.01 [0.95 – 1.07] |  |  |
| Meta analysis: no seasons, no pneumo | 0.75 [0.59 – 0.91] | 1.05 [1.01 – 1.09] |  |  |
| Meta analysis: no seasons, yes pneumonia | 0.87 [0.69 – 1.05] | 1.06 [1.01 – 1.11] |  |  |

Meta analysis plots:

Mg


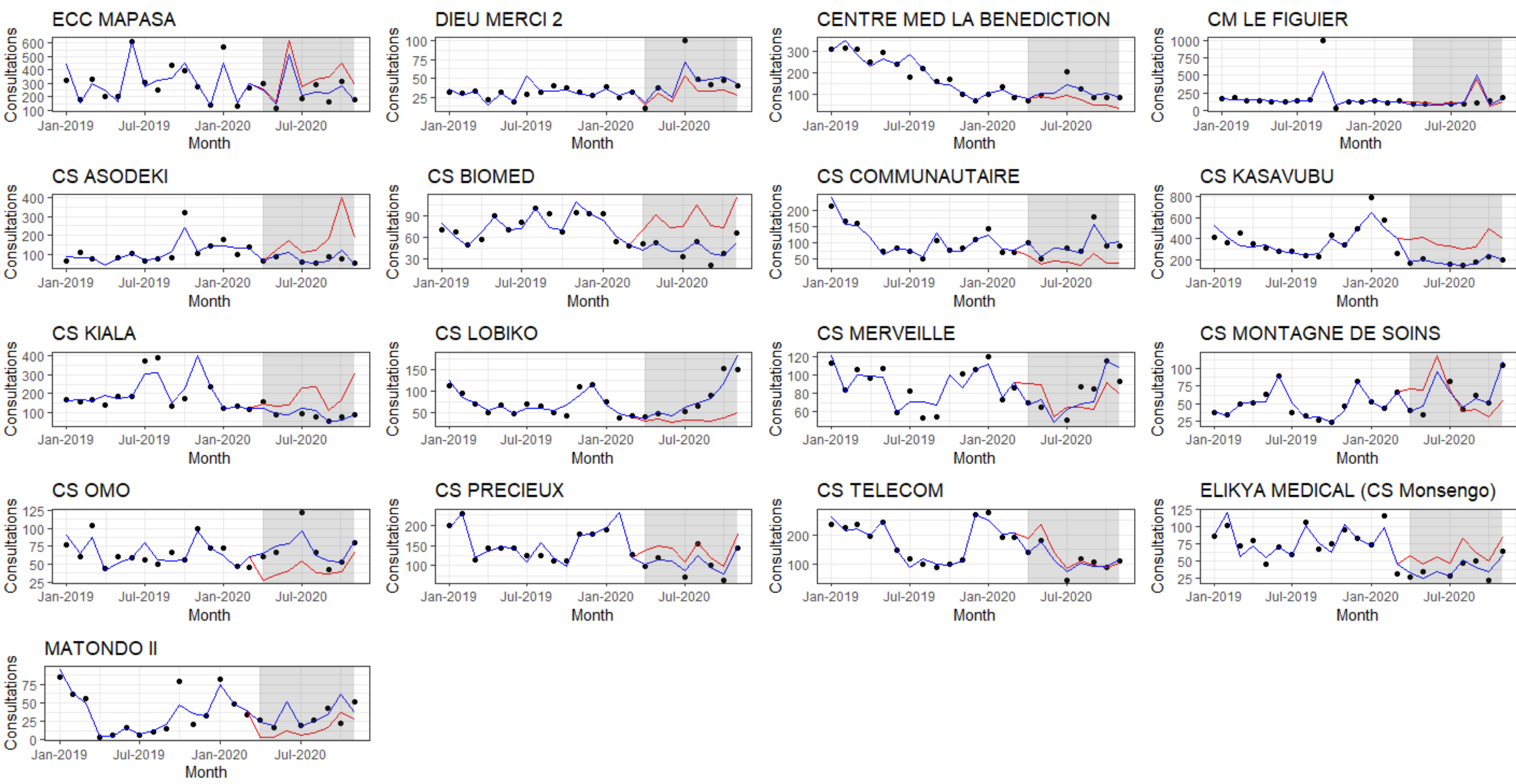


**Gombe:**

There are 20 health facilities that remain in the dataset after we remove those with more than 25% missing

We consider additional model that fits the lockdown period separately. In neither of the models are the terms for lockdown significant.

For Meta Analysis, we remove Yadah because it’s missing consecutive months of data for COVID period.

Results


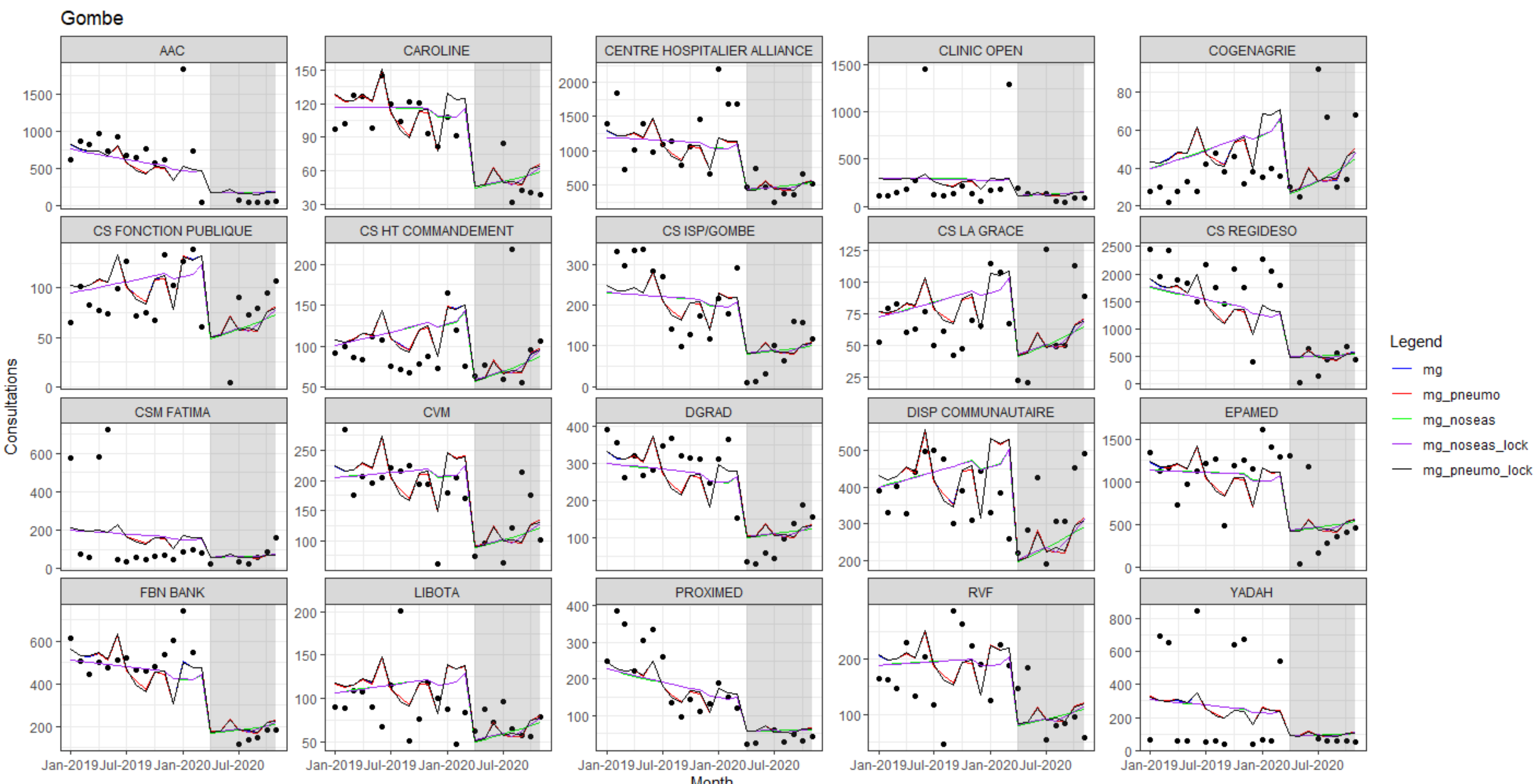


| **Model** | **IRR immediate effect** | **IRR change in slope** | **IRR immediate effect post-lockdown** | **IRR change in slope post-lockdown** | **AIC (df)** | **R2** |
| --- | --- | --- | --- | --- | --- | --- |
| mg | 0.36 [0.23 – 0.59] | 1.07 [0.96 – 1.19] | NA | NA | 5507 (42) | 0.786 |
| mg_noseas | 0.39 [0.26 – 0.58] | 1.04 [0.96 – 1.13] | NA | NA | 5504 (32) | 0.770 |
| mg_pneumo | 0.36 [0.23 – 0.59] | 1.07 [0.96 – 1.19] | NA | NA | 5509 (43) | 0.785 |
| mg_noseas_lockdown (with pneumo) | 0.40 [0.25 – 0.65] | 1.03 [0.82 – 1.30] | 0.71 [0.21 – 2.43] | 1.06 [0.78 – 1.44] | 5506 (34) | 0.769 |
| mg_pneumo_lock | 0.36 [0.20 – 0.66] | 1.07 [0.79 – 1.44] | 1.39 [0.26 – 7.51] | 0.95 [0.63 – 1.44] | 5512 (45) | 0.784 |
| Meta analysis: mg | 0.43 [0.27 – 0.59] | 1.16 [1.04 – 1.28] | NA | NA |  |  |
| Meta analysis: no seasons, yes pneumonia | 0.50 [0.35 – 0.65] | 1.12 [1.02 – 1.22] | NA | NA |  |  |

For lockdowns, did not converge in meta-analysis.

Similar sensitivity analyses were conducted for the other outcome indicators and could be made available if requested.
